# Prolonged time of after-sensation after experimental pain stimuli despite efficient conditioned pain modulation in patients with chronic neuropathic pain after traumatic nerve injuries in upper extremity

**DOI:** 10.1097/PR9.0000000000000908

**Published:** 2021-03-04

**Authors:** Adriana Miclescu, Marie Essemark, Mathias Astermark, Panagiota Gkatziani, Antje Straatmann, Stephen Butler, Rolf Karlsten, Torsten Gordh

**Affiliations:** Departments of a Surgical Sciences and; b Public Health and Caring Sciences, Uppsala University, Uppsala, Sweden

**Keywords:** Conditioned pain modulation, Cold pressor test, Conditioning stimulus, Test stimulus, Neuropathic pain, Quantitative sensory testing

## Abstract

As yet, there is limited research that can identify factors that differentiate between painful and nonpainful neuropathies after traumatic nerve injury. The aim of this study was to compare subjects with pain and without pain, all after operative nerve repair in the upper extremities.

**Methods:**

Subjects in both groups (pain, n = 69; painless, n = 62) underwent clinical assessment of sensory nerve function and psychophysical tests: quantitative sensory testing and conditioned pain modulation (CPM). Conditioned pain modulation was assessed by pain ratings to 120 seconds pressure stimuli administered before and after a 60 seconds noxious 4°C cold conditioning stimulus (CS). Time of recovery (time off) of pain intensity from peak VAS_maxc_ after CS was recorded. Questionnaires about the quality of life (RAND-36) and disability of the extremity (QuickDash) were completed.

**Results:**

There were no significant differences between groups for CPM (*P* = 0.19). Time off was 42 seconds in subjects with pain in comparison with 28 seconds in those without pain (*P* < 0.0001). Compared with individuals reporting no pain, participants with neuropathic pain after nerve injuries had 1.8 times the odds of recovering later after CS, gain of function findings at sensory examination (*P* < 0.0001), lower scores of the physical component of RAND-36 (*P* < 0.0001), and increase arm disability (*P* < 0.0001). Hyperesthesia to cold pain stimulation (*P* = 0.03) and lowered pain pressure threshold (*P* = 0.01) were found in the pain group.

**Conclusion:**

Recovery after the pain induced by cold CS indicates changes in central processing of pain and provides a potential measurement of endogenous pain modulation in individuals with chronic neuropathic pain.

## Abstract 2

Prolonged time of after-sensation after experimental pain stimuli despite efficient conditioned pain modulation was observed in patients with neuropathic pain after traumatic nerve injuries

## 1. Introduction

Why some patients are moderately affected, having no pain after a traumatic nerve injury, whereas others having a similar nerve lesion, develop debilitating chronic neuropathic pain continues to be a mystery. As yet, there is limited research that can identify factors that differentiate between painful and nonpainful neuropathies after traumatic nerve injury. In a previous study, we have demonstrated that about 50% of the patients having a traumatic nerve injury reported chronic pain after their injury, whereas the other half reported no pain. Major nerve injury, younger age, and less time from surgery were predictors for pain after traumatic nerve injuries in the upper extremities.^26^ Unfortunately, earlier studies could not detect a specific pattern using quantitative sensory testing (QST) or structural parameters using skin biopsy that would separate the neuropathy patients into painful and nonpainful individuals.^18,21,23,25,38^

Impaired conditioned pain modulation (CPM) is an indication of altered descending inhibitory modulation of pain^4,39^ and offers the opportunity to test whether patients with neuropathic pain have signs of increased pain sensitivity or abnormal CPM reactions compared with patients with neuropathy without pain. The use of the CPM paradigm in research is reported to be highly relevant for understanding persistent chronic neuropathic pain.^11,40,41^ Clear evidence of CPM deficiency^2^ has been demonstrated in central neuropathic pain because of spinal injury^1^ and in different types of peripheral neuropathic pain, including diabetic neuropathy and postherpetic neuropathy.^31–33^ The data are conflicting, because in other studies with peripheral neuropathic pain such as diabetic neuropathy^12^ or peripheral traumatic nerve injuries,^7^ no evidence of deficient CPM was identified.

Could we find some patient-related factors that characterize the 2 different outcomes, helping us to better understand why this occurs? In this study, we have investigated the role of endogenous pain modulation by comparing the variations of the CPM effect as a possible explanatory factor for the differences between painful and nonpainful neuropathy.

To the best of our knowledge, this study is the first that uses a CPM paradigm to explore potential differences in mechanisms between subjects with painful traumatic neuropathies and painless neuropathies.

## 2. Participants and methods

This study was performed at the Multidisciplinary Pain Center, Uppsala University Hospital, Sweden. The study was performed in accordance with the ethical principles for medical research involving human subjects that have their origin in the updated Declaration of Helsinki, and the study was approved by the Regional Ethics Committee (approval no: approval for the study was granted by the Regional Ethics Board in Uppsala. Project identity: ICONSS, Dnr: 2015/265; NCT03174665 for Clinical trial organization). Informed consent was obtained from all participants.

### 2.1. Patient recruitment

The patients in this cross-sectional study were recruited from a larger cohort (n = 669) study with a definite nerve injury in the upper extremities verified and described by the surgeon at the time of surgery for nerve suture or repair.^26^ They had previously answered a questionnaire about cold intolerance, pain intensity, previous medication, and the self-report Leeds Assessment of Neuropathic Signs and Symptoms (S-LANSS questionnaire).^26^ The participants with pain and S-LANSS ≥12 (indicating predominantly neuropathic pain)^5^ were recruited for the group with neuropathic pain (group A) and those without pain for the group with painless neuropathy (group B). Eligibility for participants was determined only after completion of a health history questionnaire, interview about pain intensity, and a routine clinical neurological examination which confirmed neuropathic pain or painless neuropathy. The inclusion criteria for the participants were as follows: age ≥18 years, no acute illness or diseases that might impact laboratory performance. The exclusion criteria were as follows: presence of polyneuropathy, diabetes mellitus, peripheral vascular disease, history of malignant disease, and chronic alcohol consumption. All participants were asked to refrain from any pain medication for at least 12 hours before the experimental session. The confirming sensory impairment on examination of the somatosensory system with pain in the innervation territory of a previous intraoperatively verified injured nerve and strongly indicated the diagnosis “definite neuropathic pain” for all the patients in group A.^10,37^ The subjects with sensory impairment in the innervation territory of the injured nerve but without pain were included in group B.

### 2.2. Procedures

All participants were informed about the test program after arrival in the laboratory. Participants attended a single appointment. All sessions followed the same procedure and were performed by the same trained examiner who read from a standardized instruction protocol when performing CPM.

### 2.3. Clinical assessment

The participants completed extensive questionnaires which included sociodemographic data, education level, work status, family and medical history, and time from operation. The body mass index was calculated using the formula weight/height^2^ (kg/m^2^). Baseline brachial resting blood pressure was examined before the experiment was started. All participants were found to be normotensive (111.4 ± 8.5/63.5 ± 50.0 mm Hg).

### 2.4. Pain assessment and clinical examination

Participants were asked to rate their mean clinical pain over the past week on an 11 point numeric rating scale (NRS). During clinical examination of the somatosensory system, touch was tested with a camel-hair brush (0.5 Somedic, Sweden), pain with a sharp tooth pick, and cold and warm temperature stimuli with warm (40°C) and cold (25°C) rollers (Senselab Rolltemp, Somedic). The contralateral uninjured side served as within-subject control.

### 2.5. Quantitative sensory testing

All the subjects were examined by thermal QST in the painful or nonpainful area of the innervation of injured nerve and also the corresponding area contralateral to the injured extremity. For all thermal tests, a thermode (Modular Sensory Analyser; Somedic Sales AB, Hörby, Sweden) with an area of 12.5 cm^2^ was used. The initial temperature was 32°C, and cut-off temperatures were 10 and 50°C. Warm detection thresholds (WDTs), cold detection thresholds (CDTs), heat pain thresholds, cold pain thresholds (CPTs), and ability to detect thermal and a stimulus–response–function for pinprick sensitivity (mechanical pain sensitivity [MPS]) were calculated as a mean of the 3 measurements on the injured site and noninjured control site in both groups. The 32°C baseline temperature decreased or increased at a rate of 1.0˚C/second and was stopped by the subject pressing the response button at the moment that warm or cold sensation was perceived. The mean thermal thresholds of 3 stimuli were calculated. The mechanical pain threshold (MPT), MPS including thresholds for pinprick (mechanical detection thresholds [MDT]), pain summation to repetitive pinprick stimuli (wind-up ratio), and ability to detect thermal and a stimulus–response–function for pinprick sensitivity (MPS) parameters were assessed using pinprick stimuli (MRC Systems GmbH, Heidelberg, Germany). Vibration detection thresholds were also tested. Somatosensory phenotypes were determined according to the standardized protocol of the German Research Network Neuropathic Pain.^35,36^

### 2.6. Conditioned pain modulation

**Figure 1.**
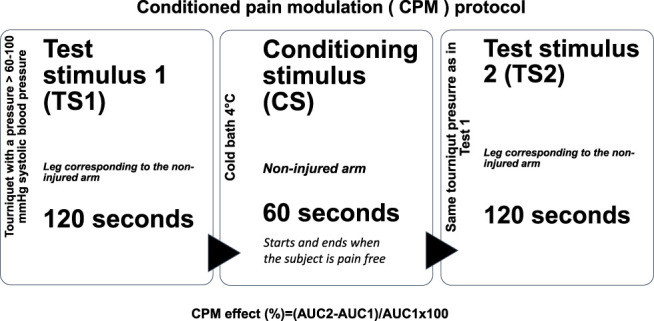
Conditioned pain modulation (CPM) protocol.

The CPM paradigm (Fig. 1) involved tourniquet pressure test stimulus (TS) applied to one leg, before and after thermal conditioning stimulus (CS) by immersion the uninjured hand in 4°C cold water.

#### 2.6.1. Test stimulus

The TS was delivered by a tourniquet applied midcalf on the leg corresponding to the noninjured arm and inflated from 60 mm Hg to 100 mm Hg above the systolic blood pressure until the pain intensity (typically 220–250 mm Hg) reported by the patient was over 50 on a 0 to 100 visual analogue scale (VAS). Nociceptive test stimuli were applied for a duration of 120 seconds before (TS1) and after (TS2) the CS.

#### 2.6.2. Conditioning stimulus

**Figure 2.**
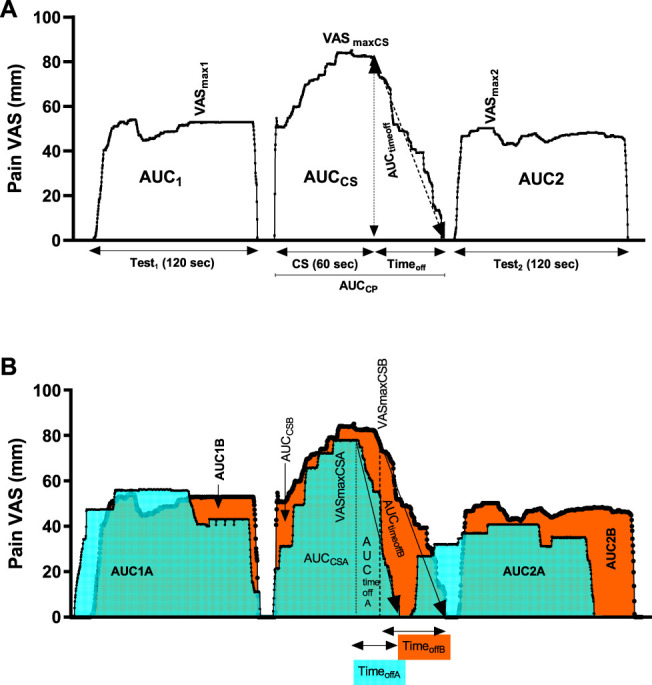
(A) Visual analogue scale (VAS) over time values expressed as area under the curve (AUC) under test stimulus 1 (AUC_1_) and 2 (AUC_2_) and under conditioning stimulus (AUC _CS_). Time off is time from peak stimulus intensity VAS_maxC_ until the decrease of pain intensity to baseline. (B) Area under the curve AUC _timeoff_A for group A (orange) and AUC_timeoffB_ for group B (blue). Longer time off for subjects with neuropathic pain (A-orange) was observed in comparison with those with painless neuropathy (B-blue) (*P* < 0.0001). CS, conditioning stimulus.

The conditioning stimuli was given by letting subjects immerse their noninjured hand up to the wrist in a cold-water bath at 4°C cooled by a refrigerated water circulator (Somedic, 2015, Sweden) for maximally 1 minute. The CS was applied after TS1 and ended when the patient withdraw the hand from the cold water bath, or maximally for 1 minute. The water level was set at a height of 7 cm, to keep the stimulated area consistent. Time in the cold-water bath (time CS) and time until the pain intensity decreased and the patients became pain free (time off) after removing the hand from cold water were expressed by the area under the curve (AUC_CS_, AUC_time_ _off_) (Fig. 2).

Immediately after the subjects became pain free after CS, an identical test stimulus (TS2) was repeated. The subject was instructed to rate continuously the pain intensity level of the both TS and CS with an electronically eVAS slider until they became pain free. They could terminate the trial at any time if they could not tolerate the painful pressure (120 seconds) or cold water (60 seconds).

#### 2.6.3. Calculations of conditioned pain modulation

To quantify CPM, the deviations of pain ratings from the set point were continuously recorded and summed over time to produce an AUC value. From the start point of the first TS forward, this dependent variable (AUC) of the VAS response over time was calculated for both TS (AUC_1_, AUC_2_) and CS (AUC_CS_). Thus, CPM was calculated as the difference in AUC of pain rating responses between the last TS after CS and the TS before CS (∆AUC = AUC_2_ − AUC_1_).

The CPM effect (%CPM) is defined as the percent change of the pain intensity evoked by TS induced before and after CS. The formula usually used is as follows: the CPM effect change was (TS1 pain subtracted from TS2 pain) divided by TS1 pain or [(TS2 pain − TS1 pain)/TS1 pain] × 100. The percentage of CPM (%CPM) = ∆AUC × 100/AUC_1_. The CPM effect varies from pain inhibition to facilitation. Therefore, negative CPM scores indicated pain inhibition and positive CPM scores indicated pain facilitation. Efficient CPM was defined as the ability of the individuals to inhibit at least 29% of pain.^34^

### 2.7. Measures

Some measures were completed by the subjects before coming to the study such as S-LANSS (Leeds Assessment of Neuropathic Symptoms and Signs).^5^

### 2.8. Quality of life

Quality of life was measured at the start of the study with the 36-Item Short-Form Health Survey (RAND-36) a health survey that laps 8 concepts investigating physical and mental status.^17^

### 2.9. Depression and anxiety

The Hospital Anxiety and Depression Scale (HADS) questionnaire was given to both experimental groups to identify the grade of anxiety disorder and/or depression. The total score for each domain was calculated as the sum of the respective 7 items (ranging from 0 to 21), with normal values (0–7), borderline cases (8–10), and abnormal cases (11–21).^45^

### 2.10. Quick disability of the shoulder, arm, and hand

QuickDash is a short, reliable, and valid measure of physical function and symptoms related to upper-limb musculoskeletal disorders by shortening the full, thirty-item (Disabilities of the Arm, Shoulder, and Hand [DASH]) Outcome Measure.^3^

The last 3 questionnaires were completed when the participants came to the experiment.

## 3. Statistical considerations

All statistical analyses were performed with IBM SPSS Statistic version 19.0.0.1, GraphPad Prism 8, and SAS version 9.4 (SAS Institute, Inc). Descriptive statistics are presented as means and standard deviations for continuous variables and absolute numbers and percentages for categorical variables. For continuous variables, the Mann–Whitney *U* test was used. To compare CPM between subjects with neuropathic pain and painless neuropathy and to compare differences between the subjects in the same group, a 2-way analysis of variance (group and side) was performed. A post-hoc unpaired *t* test was performed for between group comparisons and a post-hoc paired *t* test for within-group comparisons. For AUC_timeoff_ and time off, we estimated proportional odds models (cumulative probability models using the link function) including the following variables: group (A vs B), age, gender, VAS MAX c, and duration in the cold-water bath. To assess correlation between pain adaptability and pain modulation, Spearman correlation coefficients were used. Most QST-parameters (CDT, WDT, MDT, CPT, and WPT) were log-transformed to conform to a normal (Gaussian) distribution. Quantitative sensory testing was standardized using z-transformation, with z-values above 0 indicating lower thresholds and gain of function, whereas z-scores below 0 indicate hyposensitivity.^35,36^ The level of significance was set at a *P*-value <0.05.

## 4. Results

### 4.1. Study participants, RAND-36, Quick Disability of the Shoulder, Arm, and Hand, hospital anxiety and depression scale

Of 669 subjects previously screened with S-LANSS after traumatic nerve injury, and treated with nerve suture or nerve repair,^26^ 146 of them received an invitation, and 131 fulfilled the criteria by participating in all tests. Sixty-nine (30 women) subjects had neuropathic pain [group A, 48 years], and 62 (24 women) subjects had a painless nerve lesion [group B, 49 years]. Both groups included subjects with the same types of nerve injuries localized to digital, radial, median, ulnar nerves, and also multiple nerve lesions (*P* = 0.643). All the subjects in group A had S-LANSS score ≥12, and all reported pain. Group B had S-LANSS <12. Of the subjects with pain resulting from traumatic nerve injury, 80% had no pain medication despite the presence of rather severe pain (NRS ≥ 6) in 72% of the patients (50/69). Only 15% of subjects with chronic neuropathic pain considered that the medications they had tried had any effect on their pain (Table 1).

**Table 1:** Demographic and clinical characteristics of subjects.

Measure	Group A (n = 69) neuropathic pain		Group B (n = 62) non painful neuropathy		*P*
	Value	%	Value	%	
Age, y					0.443
Median	48 (20–86)		49 (19–83)		
Gender					
Male/female (N)	39/30	76.9	38/24	63.1	0.545
Time from nerve suture operation, y					
Mean ± SD	1.1 ± 2.5		2.3 ± 1.3		**<0.001**
BMI					
Mean ± SD	26 ± 5.1		26 ± 3.9		0.397
ASA physical status					0.642
I	35	50.8	31	50	
II	30	43.4	25	40	
III	4	5.8	6	10	
Employment (n)					0.461
Employed	49	71	50	80.6	
Retired	10	14.5	7	11.2	
Unable to work	9	13	3	4.8	
Others	1	1.5	2	3.2	
Nerve injury (n)					0.643
Digital nerves total (median, ulnar, radial)	31 (15, 10, 5)	44.9	41 (17, 8, 15)	66.1	
Median	9	13	3	4.8	
Ulnar	6	8.6	1	1.6	
Radial	3	4.3	4	6.4	
Multiple nerves	20	30.4	13	20.9	
Reoperation (n)	11	15	4	6.4	0.333
Dominant hand (right)					
Right	57	82.6	56	90.3	0.957
Injury site (right)					
Right	28	40	27	43	0.853
Pain intensity (NRS 0–10)					**<0.0001**
Maximum last week	6.5 ± 2.5		0.01 ± 0.1		**<0.0001**
Minimum last week	1.7 ± 1.8		0		**<0.0001**
Average last week	4.1 ± 2.1		0.18 ± 0.25		**<0.0001**
Current	3.2 ± 2.7		0		**<0.0001**
Other chronic pain	34	49.2	21	33.0	0.064
Joint pain	18	26	16	25	
Low back pain	12	17.3	6	9.6	
Headache	4	5.7	2	3.2	
Others	9	13	1	1.6	
S-LANSS					
Mean ± SD	19.2 ± 3.4		4.3 ± 3.1		**<0.0001**
LANSS part A					
Mean ± SD	11.4 ± 2.7		0.3 ± 1.7		
LANSS part B					
Mean ± SD	6.8 ± 2.0		3.9 ± 2.7		
HADS anxiety					0.133
0–7 points (normal values)					
No anxiety	47	68.1	50	80.6	
8–10 (borderline cases)					
Mild anxiety	12	17.3	8	12.9	
≥11–21 (abnormal cases)					
Severe anxiety	10	14.4	4	6.4	
HADS depression					0.143
0–7 points (normal values)					
No depression	60	86.9	53	85.4	
8–10 (borderline cases)					
Mild depression	6	8.6	7	11.2	
≥11–21 (abnormal cases)					
Severe depression	3	4.3	2	3.2	
QuickDASH (mean ± SD)	34 ± 22		7.6 ± 12		**<0.001**
RAND-36 (mean ± SD)					**<0.001**
Physical function (PF)	77 ± 20		89 ± 17		**<0.0001**
Physical role/function (RP)	53 ± 37		83 ± 29		**<0.0001**
Pain (BP)	52 ± 22		83 ± 23		**<0.0001**
General health (GH)	67 ± 25		74 ± 19		0.149
Physical component RAND-36	251 ± 85		330 ± 69		**<0.0001**
Mental health (MH)	77 ± 21		79 ± 18		0.663
Emotional role/function (RE)	68 ± 38		83 ± 32		0.098
Social function (SF)	79 ± 26		87 ± 22		**0.040**
Vitality (VT)	62 ± 24		64 ± 20		0.429
Mental health component RAND-36	285 ± 99		313 ± 81		0.195

ASA American Society of Anesthesiology physical status; BMI, body mass index; y, years. Bold font indicates statistical significance.

### 4.2. RAND-36, Quick Disability of the Shoulder, Arm, and Hand, hospital anxiety and depression scale

No difference was observed between the experimental groups for either anxiety or depression scores measured with the HADS questionnaire (Table 1). There were statistically significant reductions of the physical component of RAND-36 scores (*P* < 0.0001) in the patients with neuropathic pain related to a decrease of physical function (*P* < 0.0001), physical role function (*P* < 0.0001), and pain (BP) (*P* < 0.0001). No difference was observed between groups related to the mental health component of RAND-36 (*P* = 0.195). The average Quick DASH survey scores were 33.46 for subjects with pain and 7.5 for subjects without pain (*P* < 0.0001). The results indicated a higher degree of disability in patients with neuropathic pain.

### 4.3. Sensory abnormalities after standard bedside examination

**Figure 3.**
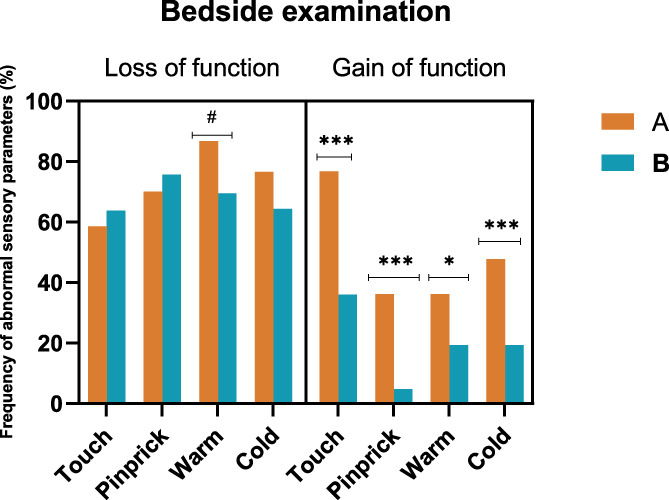
Bar graph of the frequency of abnormal sensory parameters (%) found at bedside examination for the subjects included in the group with neuropathic pain (orange) and for those with painless neuropathy (blue). The loss of function describes subjects with decrease and loss of sensation to touch, pinprick, warm and cold and gain of function represents dynamic mechanical allodynia and hyperesthesia to touch, cold and warm hyperesthesia. Gain of function (P*** < 0.0001) evidenced by dynamic mechanical allodynia, cold hyperesthesia, pinprick hyperalgesia, but also loss of function to warm sensation (*P*# = 0.03) were the sensory abnormalities seen in subjects with pain in comparison with those with nonpainful neuropathic injuries.

Gain of function (*P* < 0.0001) evidenced by dynamic mechanical allodynia (*P* < 0.0001), cold hyperesthesia (*P* = 0.0009), pinprick hyperalgesia (*P* < 0.0001) (Fig. 3), but also loss of function to warm sensation (*P* = 0.03) were the sensory abnormalities seen in subjects with pain in comparison with those with nonpainful neuropathic injuries.

### 4.4. Quantitative sensory testing

**Figure 4.**
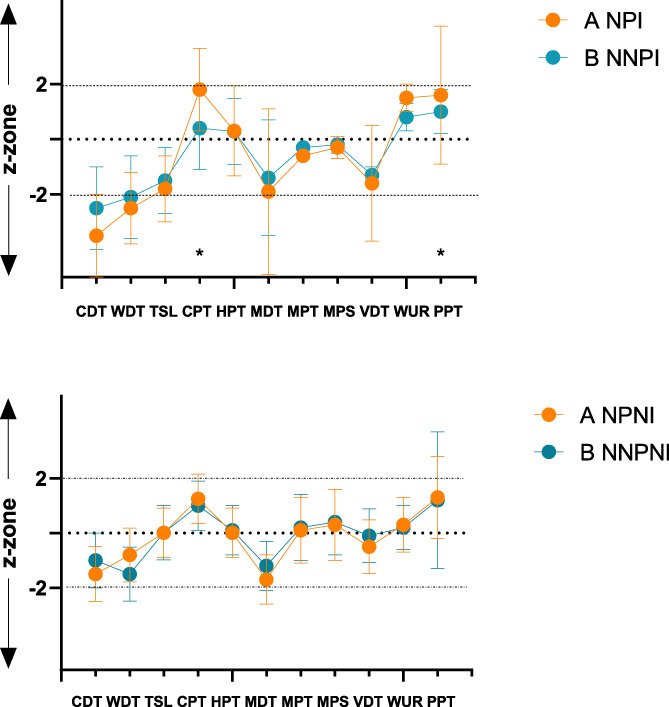
Quantitative sensory testing. The orange symbols represent QST for subjects with neuropathic pain group A (neuropathic pain injured [NPI] and noninjured hand [NPNI]) and the blue symbols for subjects with neuropathy without pain group B (without neuropathic pain injured [NNPI] and without neuropathic pain noninjured hand [NNPNI]). Data are the population mean z-scores. Z-scores were calculated in relation to a population of healthy subjects as determined by Rolke et al.^35,36^ The horizontal solid lines indicate the +2 and −2 z-score boundaries. A specific QST test is considered abnormal if the test-value lies above the upper or below the lower boundary. Significant differences between group A and B injured hands were seen for CPT (*P* = 0.03) and PPT (*P* = 0.01), but no difference between noninjured hands were found as seen in the second graph. The differences between injured and noninjured hand were presented in text. CDT, cold detection threshold; CPT, cold pain threshold; HPT, heat pain threshold; MDT, mechanical detection threshold; MPS, mechanical pain sensitivity; MPT, mechanical pain threshold; PPT, pressure pain threshold; QST, quantitative sensory testing; TSL, thermal sensory limen; VDT, vibration detection threshold; WDT, warm detection threshold.

When the territory of innervation of the injured nerve in subjects with pain was compared with the contralateral healthy side, there were observed thermal differences for CDT, WDT, CPT, TSL (*P* < 0.0001), but not for heat pain thresholds (*P* = 0.08). Elevated mechanical (MDT; *P* < 0.001), lowered PPT (*P* < 0.05) and MPT, vibration detection thresholds (*P* > 0.05 each) were seen. In the individuals with neuropathy without pain, cold detection thresholds (CDT; *P* < 0.0001; CPT; *P* < 0.001), MDT (MPT; *P* < 0.01, each) were elevated compared with the contralateral side and less ability to detect thermal changes were seen (TSL; *P* < 0.05). Except for statistically significant differences in CPT (*P* = 0.03) and lowered PPT in the pain group (*P* = 0.01), no other differences in QST were observed between the subjects with pain and painless neuropathy (Fig. 4).

### 4.5. Conditioned pain modulation and conditioned pain modulation effect

**Figure 5.**
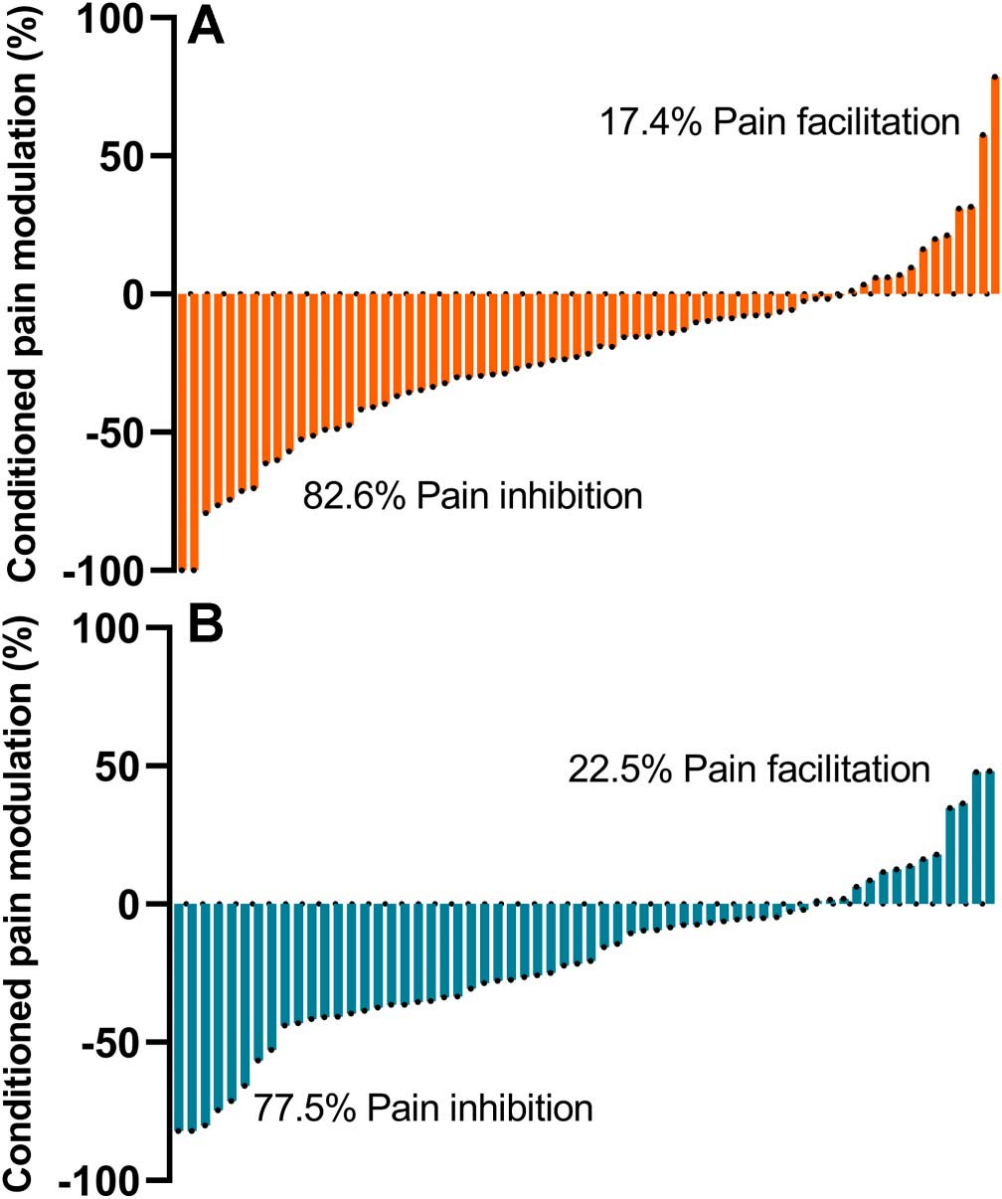
Individual pain inhibition or pain facilitation occurring during the conditioned pain modulation paradigm in subjects with neuropathic pain (orange) and subjects with neuropathy without pain (blue).

The CPM effect or pain inhibition was present in 57 subjects (82%) in group A and 48 subjects (77%) in group B. A CPM effect> 29% indicating a significant analgesic response during CPM^34^ was seen in 28 (40%) of the subjects with neuropathic pain and 24 (39%) subjects with neuropathy without pain. A deficit in inhibitory CPM efficacy or pain facilitation was observed in 12 (17%) subjects in group A and 14 (22%) subjects in group B (Fig. 5). There were no significant differences in AUC1 and AUC2 between groups (*P* > 0.05).

### 4.6. Time for pain to wean off after conditioning stimulus and the response to conditioning stimulus

In group A, 46 (66%) of the subjects could leave their hands in cold water for the full 60 seconds and in group B, 35 (54%) of subjects. Pain after CS lasted longer in patients with neuropathic pain (42 seconds) in comparison with those without pain (19 seconds) (*P* < 0.0001).

As illustrated in Table 2, there were no statistically significant differences in CPM (*P* = 0.19), but significant differences were seen regarding AUC time off (*P* < 0.0001), time off (*P* < 0.0001), and AUC_CS_ (*P* < 0.0001) between the neuropathic pain and neuropathy without pain groups. After adjusting for age, gender, VAS MAX c, and duration in bath with a cumulative logit model, significant difference between groups A and B, for time off (*P* = 0.04) was seen. The odds ratio of a subject in the group with pain recovering later after CS was 1.8 (95% CI 0.97–3.29) times the odds of a subject with painless neuropathy (Fig. 2).

**Table 2:** Differences in endogenous pain modulation between the subjects with neuropathic pain (A) and nonpainful neuropathy (B).

Group	N Obs	Variable	Median	25th Pctl	75th Pctl	Mean	Std Dev	CI 95%	CI 95% diff	*P*
A	69	AUC_timeoff	1942.5	832.6	2828.9	2169.4	1803.2	1726 to 2603	243 to 1126	**<0.0001**
B	62	AUC_timeoff	1398.0	759.5	2078.4	1531.8	981.4	1235 to 1724		
A	69	Time off	36.0	18.6	50.7	42.6	45.9	31.6 to 52.7	20 to 42	<0.0001
B	62	Time off	26.8	15.7	36.1	28.5	16.5	23.9 to 32.2		
A	69	AUC_1_	5877	4192	8933	6148	3211	5374 to 6971	−653 to 230	0.184
B	62	AUC_1_	6063	4603	8591	6349	2982	5602 to 7116		
A	69	AUC_2_	4597	2219	6477	4011	3219	4019 to 5466	−972 to −88.9	0.987
B	62	AUC_2_	4755	2729	6655	4990	3098	4442 to 6104		
A	68	∆-AUC	−1284	−2698	−228	−1450	2245	−1993 to −906	−807 to 79	0.214
B	62	∆-AUC	−623	−2251	−55	−1086	2014	−1578 to −504		
A	69	CPM effect	−23	−21	−4.2	−22	33	−29.9 to −14.3	−381 to 376	0.615
B	62	CPM effect	−21	−37	−2.5	−19	29	−27.2 to −12.1		
A	69	VAS max1	67	37	64	56	26	49 to 62	−43 to 44	0.364
B	62	VAS max1	49	36	66	54	24	47 to 60		
A	69	VAS max2	42	21	61	44	26	37 to 50	−44 to 44	0.567
B	62	VAS max2	42	24	63	45	26	38 to 51		
A	69	VAS maxCS	55	42	70	87	22	51 to 61	1276 to 13	0.053
B	62	VAS maxCS	55	47	67	84	17	67 to 80		
A	69	AUC CS	4448	2883	6379	4785	2533	1736 to 2603	351 to 1235	**<0.0001**
B	62	AUC CS	3843	2369	5388	3991	1868	3517 to 4667		
A	69	Duration CS	82	64	103	88	49	76 to 100	−45 to 44	0.246
B	62	Duration CS	73	57	89	73	44	67 to 80		
A	69	Duration bath	53	30	60	48	17	42 to 50	11 to 69	0.147
B	62	Duration bath	51	32	59	45	17	41 to 50		

VAS max CS, is the maximum pain intensity on the visual analog scale; AUC_CS_ area under the curve = pain ratings in time under CS; duration CS (sec) total time of conditioning stimulus; duration in bath-seconds for hand in bath. Bold font indicates statistical significance.

%CPM = = ∆AUCx100/AUC_1_; ∆-AUC, AUC_2_-AUC_1_; AUC time off, area under the curve from maximum pain intensity over time until the subjects became pain free; AUC1, area under the curve test 1; AUC2, area under the curve test 2; CPM, conditioned pain modulation; CS, conditioning stimulus; VAS max1, maximum visual analogue scale test 1; Time off is time from maximum pain intensity to 0 after conditioning stimulus; VAS, Visual Analogue Scale.

No statistical differences were seen between mean peak VAS (VAS_CS1_ = 87.4 ± 17; VAS_CS2_ = 84.3 ± 22) during CS between groups (*P* = 0.46) and no correlation between time off and peak VAS was seen for all the subjects (*P* = 0.73, r = −0.03), as well for group A (*P* = 0.39, r = −0.9).

## 5. Discussion

In contrast to our working hypothesis, we found that neuropathic pain patients had a normal function of some aspects of endogenous pain modulation, as no differences in CPM was observed in comparison with subjects with neuropathy without pain. A new finding in this study was that the neuropathic pain patients needed a longer time to recover from the pain experience induced by the conditioning pain stimuli.

### 5.1. Conditioned pain modulation

The potential mechanisms that produce and exacerbate chronic pain remain relatively unknown.^6^ Conditioned pain modulation has gained considerable attention among the potential mechanisms that produce pain, primarily because of reports that reduced CPM is associated with the presence of several chronic pain conditions.^9,13,29,30^ There are doubts as to the validity of CPM as a biomarker helping us understanding the mechanisms of clinical pain^9^ supported by the fact there is no significant correlation between CPM and clinical manifestations of chronic pain. A large proportion of both subjects with neuropathic pain and those with painless neuropathy experienced less pain during the second application of the TS after the administration of CS. However, in both groups, a small proportion of subjects experienced pain facilitation during CPM. The results clearly demonstrated that the presence of CPM cannot be used as a mechanistic sign in differentiating chronic neuropathic pain from neuropathy without pain in this study group. The results underline the speculation presented in another study published by Granovsky^12^ that the patients with neuropathic pain (in that case diabetic neuropathy pain) seemed to have “normalized” CPM with the chronicity of the pain syndrome.

The patients in this cross-sectional study was taken from a larger cohort examining risk factors associated with chronic neuropathic pain after nerve trauma followed by surgical repair.^26^ All the patients had a definite traumatic nerve lesion, in detail described by the surgeon, who has seen the injured nerve intraoperatively, even if confirmatory tests for nerve lesion, as required for this diagnosis according to the present definition^10,37^ were not performed. After all, the “confirmatory tests,” such as neurophysiological tests or imaging, are only indirect, “proxy” methods to detect a nerve lesion. The large proportion of subjects with efficient CPM found in both groups might be explained by the small percentage of subjects with severe anxiety, depression, or subjects taking pain medication, factors known to be related with CPM response.^29,43^ Most important is the fact that the nerve injuries present after trauma involved the same type of nerves all located in the upper extremities. This decreased the potential for confounding factors in pain etiology and reduced the risk of bias in the conduct and reporting CPM reliability. There are methodological discrepancies among different laboratories in inducing CPM^34^ and ambiguous results. Currently, except for some practical recommendations,^42^ there is no description of a standardized CPM. Concerning the reliability of our protocol for the assessment of CPM, it was demonstrated previously that cuff algometry for TS and cold bath for CS, the use of sequential protocol testing are appropriate to be used in CPM assessment.^14,42^

### 5.2. Time off and response to conditioning stimulus

It has long been known that changes in noxious stimulus intensity have an effect on painful after sensations,^16^ but the response after a noxious cold stimulus has not been characterized. It was known that an analgesic mechanism known to quantify endogenous pain modulation is activated during noxious stimulus offset.^15^ Among other methods of measuring endogenous pain modulation, one can examine offset analgesia (OA)^7,44^ which refers to disproportionately large decreases in pain ratings evoked by small decreases in stimulus intensity. In patients with neuropathic pain,^30^ OA was found to be attenuated. If the OA magnitude represents the descending segment of pain sensation after a decrease of noxious stimulus, the slower speed of recovery from maximum pain intensity might also be used as a marker of deterioration of descending modulation.^24^ Thus, similar to the CPM and OA paradigm, the recovery time after a conditioning cold stimulus can also be used to quantify endogenous pain modulation. Similarly to evidence for a smaller effect of OA^24,30^ in chronic neuropathic pain patients^24^ as compared with healthy controls, it seemed that the delayed recovery time after a painful stimulus distinguished subjects with neuropathic pain from nonpain subjects. Both CPM and recovery time indicate endogenous pain modulation, but the different results obtained here probably depended on the different neural mechanisms involved in CPM and recovery time.^7^ The mechanisms of different endogenous pain modulation pathways differ with probably more brain-driven pain modulation during recovery after cold CS, because of activation of brain regions such as periaqueductal gray, dorsolateral prefrontal cortex, insula, medulla, pons, and cerebellum.^28,44^

### 5.3. Quantitative sensory testing

In comparison with the studies on healthy individuals without nerve injuries,^8,20^ a large percent of the study participants in our study, who immersed the contralateral arm in the cold bath could not tolerate a 1 minute duration of CS in 4 C water. Cold intolerance is a relatively common symptom after peripheral nerve injury^19,26^ and is interpreted as a hyperexcitability in the pain system at the location of neuropathic pain.^28^ Except for a predominantly gain of function at bedside examination and elevated values for CPT and lower PPT in subjects with neuropathic pain,^18^ no other differences between subjects with pain and painless neuropathy were observed using the same QST protocol. In a recent study, Müller et al.^27^ found that hypersensitivity with cold stimulation and lower pressure pain thresholds suggested alterations in central pain processes and were associated with poor recovery after surgery. The presence of later recovery after cold stimulus and reduced tolerance for the cold pressor test^20^ in the contralateral side of nerve injury, indicated changes in central processing of pain which may be important contributors to the development of chronic neuropathic pain.^22^

### 5.4. Limitations of the study

First, because this sample of subjects was drawn from the subjects with traumatic nerve lesions of the wrist and hand, and in addition operated with nerve suture in a hand surgical setting, the severity and comorbidity findings may be different from that seen in patients with other causes of chronic neuropathic pain. Quantitative sensory testing and CPM data were compared between subjects with neuropathic pain and neuropathy without pain, but not with an independent healthy control group without nerve injury. However, this fact may also be looked on as a strength. Furthermore, this study was a cross-sectional postsurgery study. On the other hand, the patients in our cohort were identified after their initial nerve trauma, and followed up regularly for several years, and all of them having a well-defined definite nerve lesion. In that perspective, the cohort is perhaps unique.

## 6. Conclusion

In contrast to our hypothesis, the neuropathic pain patients had a well-functioning CPM, not differing from that of patients with nerve lesion with no pain. A new finding demonstrated in the neuropathic pain patients was a characteristic pattern of delayed recovery time to baseline in reported pain intensity after a cold CS applied to the non-neuropathic hand. This study extends previous findings of the relationship between neuropathic and neuropathy without pain, hopefully contributing to better understanding of chronic neuropathic pain pathophysiology.

In addition, the findings may potentially provide a new way to study and measure the process of endogenous pain modulation, using cold stimuli in relation to painful “after-sensations.”

## Disclosures

The authors have no conflicts of interest to declare.
